# The Emerging Therapeutic Role of Prostaglandin E2 Signaling in Pulmonary Hypertension

**DOI:** 10.3390/metabo13111152

**Published:** 2023-11-16

**Authors:** Lan Ye, Bing Wang, Hu Xu, Xiaoyan Zhang

**Affiliations:** 1 Advanced Institute for Medical Sciences, Dalian Medical University, Dalian 116041, China; yel@dmu.edu.cn; 2 Department of Endocrinology and Metabolism, The Central Hospital of Dalian University of Technology, Dalian 116000, China; wangbing_1999_1981@163.com; 3 Health Science Center, East China Normal University, Shanghai 200241, China

**Keywords:** chronic obstructive pulmonary disease, pulmonary hypertension, prostaglandin E2, pulmonary vascular remodeling

## Abstract

Mild-to-moderate pulmonary hypertension (PH) is a common complication of chronic obstructive pulmonary disease (COPD). It is characterized by narrowing and thickening of the pulmonary arteries, resulting in increased pulmonary vascular resistance (PVR) and ultimately leading to right ventricular dysfunction. Pulmonary vascular remodeling in COPD is the main reason for the increase of pulmonary artery pressure (PAP). The pathogenesis of PH in COPD is complex and multifactorial, involving chronic inflammation, hypoxia, and oxidative stress. To date, prostacyclin and its analogues are widely used to prevent PH progression in clinical. These drugs have potent anti-proliferative, anti-inflammatory, and stimulating endothelial regeneration properties, bringing therapeutic benefits to the slowing, stabilization, and even some reversal of vascular remodeling. As another well-known and extensively researched prostaglandins, prostaglandin E2 (PGE2) and its downstream signaling have been found to play an important role in various biological processes. Emerging evidence has revealed that PGE2 and its receptors (i.e., EP1–4) are involved in the regulation of pulmonary vascular homeostasis and remodeling. This review focuses on the research progress of the PGE2 signaling pathway in PH and discusses the possibility of treating PH based on the PGE2 signaling pathway.

## 1. Introduction

Chronic respiratory diseases such as chronic obstructive pulmonary disease (COPD) are major causes of morbidity and mortality worldwide [1]. Smoking is the predominant risk factor for COPD. For a long time, the prevalence of COPD was significantly higher in men than in women [2]. PH, one of the established complications of COPD, results in an augmented right ventricular workload, leading to right ventricular (RV) failure and potentially fatal outcomes [3,4,5]. Since the first World Workshop on Pulmonary Hypertension was held by the World Health Organization in Geneva in 1973, PH has been defined as mean Pulmonary Artery Pressure (mPAP) ≥ 25 mmHg measured by right heart catheterization at rest [6]. This definition remained unchanged during follow-up meetings of the World Symposium on Pulmonary Hypertension (WSPH) from 1998 to 2013 [7]. In the updated guidelines for the diagnosis and treatment of PH in 2022, this definition was changed to mPAP > 20 mmHg at rest [4]. Pulmonary arterial hypertension (PAH), PH caused by pulmonary disease or hypoxia, chronic thromboembolic PH, and PH caused by unknown multifactorial mechanisms all belong to the category of precapillary pulmonary hypertension. Its hemodynamic characteristics are pulmonary artery wedge pressure (PAWP) ≤ 15 mmHg and PVR > 2 WU [4]. PH can be divided into mild (26–35 mmHg), moderate (36–45 mmHg), and severe (>45 mmHg) according to the mPAP at rest. The incidence of PH is second only to coronary heart disease and hypertension among cardiovascular diseases, and it represents a major global health problem due to its high mortality rate [8,9]. According to the current classification criteria, PAH can be divided into idiopathic, heritable, induced by drugs or toxins, or associated with conditions such as connective tissue disease (CTD), congenital heart disease (CHD), portal hypertension, HIV infection, or schistosomiasis. In the Western world, idiopathic PAH (iPAH) is the most common subtype of PAH [10]. The severity of clinical symptoms in patients is closely related to the decrease in cardiac output and the increase in RV pressure [11]. Galie N et al. suggested that early detection of iPAH patients can reduce the occurrence of severe right heart failure and improve quality of life [12]. Without timely and appropriate treatment, adults with PH have an average life expectancy of 2.8 years from the time of diagnosis, while children have an average life expectancy of less than 10 months [13,14]. At present, the main clinical treatment methods are prostacyclin (prostaglandin I2, PGI2) analogues, endothelin receptor antagonists, and phosphodiesterase inhibitors to reduce PAP, thereby reducing PVR and right heart stress (Table 1) [15,16,17,18]. PGI2 is currently the main drug for the treatment of PH, and a series of PGI2-based compounds (epoprostenol, iloprost, treprostinil and beraprost sodium, etc.) have been studied as well [15]. Bosentan is an orally active dual (A and B) endothelin receptor antagonist that improves PVR [12]. As a phosphodiesterase 5 (PDE5) inhibitor, sildenafil can effectively induce lung dilatation, prevent pulmonary vascular remodeling, and reduce right ventricular hypertrophy [19]. PGI2 mainly acts on two receptors: G protein-coupled IP receptors on the cell surface and peroxisome proliferator-activated receptors (PPAR-β). Nitric oxide (NO) and PGI2 synergistically maintain vascular function [20]. Emerging evidence has revealed that PGE2 and its receptors (i.e., EP1–4) are involved in the regulation of pulmonary vascular homeostasis and remodeling. This review focuses on the research progress of the PGE2 signaling pathway in PH and discusses the possibility of treating PH based on the PGE2 signaling pathway.

## 2. Pathophysiology of PH

PH is characterized by structural remodeling of the distal pulmonary artery, resulting in vessel wall thickening and lumen occlusion along with increased PVR [21,22]. Patients with PH often present with elevated pulmonary artery pressure, extensive vascular remodeling and stenosis, and right heart hypertrophy, eventually leading to right heart failure and death [23]. PH is entirely due to increased PVR. Although many factors can lead to an increase in PVR, alveolar hypoxia is the most dominant [24]. Most notably, obstructive sleep apnea syndrome and obesity–hypoventilation syndrome may increase the severity of alveolar hypoxia, thereby increasing PVR and leading to a significant rise in pulmonary artery pressure [25].

The main pathological feature of PH is occlusion of small pulmonary arteries caused by endothelial dysfunction and the uncontrolled proliferation of pulmonary artery smooth muscle cells (PASMCs) and fibroblasts [26]. The proliferation rate of cultured PASMCs isolated from patients with iPAH has been found to be nearly twice that of normal cells [27]. Pulmonary arterial endothelial cells (PAECs) regulate the contractile and diastolic function of vessels by secreting contractile factors such as thromboxane A2 (TXA2) and endothelin-1 (ET-1) as well as diastolic factors such as PGI2 and nitric oxide (NO). Gene mutation, hypoxia, drug toxicity, and other environment changes can cause endothelial injury, leading to the increase in contractile factors and decrease in diastolic factors, resulting in contraction of pulmonary vessels, elevation of pulmonary artery pressure, and eventually causing PH [28]. PAEC dysfunction plays a key role in the progression of PH; dysfunctional endothelial cells are characterized by impaired cell–cell junctions and high permeability, which allow proinflammatory factors to penetrate into the smooth muscle layer and induce abnormal proliferation of PASMCs [9]. In addition, the increase of intracellular Ca^2+^ induces the phosphorylation of myosin light chain (MLC), actin polymerization, and cytoskeleton remodeling, which all cause the contraction of PASMCs [29]. Abnormal endothelial cells (ECs) and their proliferation, differentiation, and interactions with pericytes and smooth muscle cells (SMCs) are fundamental mechanisms of many cardiovascular diseases, including PH and atherosclerosis [30]. Pericyte recruitment plays a key role in the development of PH. Studies have shown that PAEC dysfunction and upregulation of transforming growth factor-β (TGF-β) during the development of PH increase the coverage of microvascular pericytes, which differentiate into PASMCs or fibroblasts in small pulmonary arteries. The increasing coverage of microvascular pericytes promotes the remodeling of pulmonary arterioles [31].

## 3. Pulmonary Vascular Remodeling in PH

Vascular remodeling is a process involving alteration of the structure and arrangement of blood vessels through cell growth, cell death, cell migration, and production or degradation of extracellular matrix (ECM), and is involved in the development and progression of various cardiovascular diseases such as hypertension, atherosclerosis, and aneurysm. It is a critical adaptive feature for the maintenance of blood flow in vessels with thickening intimas [32,33]. Vascular remodeling is seen in large and smaller distal pulmonary arteries. Under physiological conditions, SMCs of normal mature blood vessels exist in a state of contraction, differentiation, and quiescence [34]. The proliferation of SMCs is the main pathological features of vascular remodeling. Pulmonary vascular remodeling includes processes such as endothelial dysfunction, activation of fibroblasts and PASMCs, ECM deposition, vascular wall cell-to-cell interactions, and recruitment of circulating progenitor cells [35]. A large number of inflammatory cell infiltrations can be observed around the pulmonary arteries in PH, suggesting that inflammatory cells (mast cells, macrophages, T lymphocytes, B lymphocytes, and dendritic cells) may be involved in pulmonary vascular changes [36]. Inflammatory cells and damaged PAECs and PASMCs can release a large number of cytokines and chemokines to exert chemotactic and adhesive effects, resulting in PAEC injury, PASMC proliferation, and immune cell recruitment, forming a positive feedback effect that promotes pulmonary vascular remodeling [37,38]. Growth factors act as potent mitogen and chemoattractant agents for vascular cells such as SMCs, fibroblasts, and ECs, initiating intracellular signaling cascades that lead to cell proliferation, migration, and resistance to apoptosis by binding to and activating cell surface tyrosine kinase receptors. Growth factors such as vascular endothelial growth factor (VEGF), fibroblast growth factor (FGF), epidermal growth factor (EGF), platelet-derived growth factor (PDGF), and hepatocyte growth factor (HGF) are most clearly implicated in PH [39]. In addition, excessive collagen deposition in the ECM reduces pulmonary vascular stiffness, which is the main feature of pulmonary vascular remodeling [40].

PH is a multifactorial and heterogeneous disease, with a variety of different pathogenic alterations observed in similar phenotypes: (1) environmental factors such as air pollution, chronic hypoxia and smoke exposure, altered shear stress, and infections; (2) mutations in genetic susceptibility genes such as bone morphogenetic protein receptor 2 (BMPR2), activin receptor-like kinase 1 (ALK1), voltage-gated potassium channel 1.5 (K_v_1.5), potassium channel subfamily K member 3 (KCNK3), etc.; (3) systemic or circulating factors such as hormone and iron availability, blood coagulation, and inflammation. These pathogenic alterations initially trigger endothelial dysfunction in normal pulmonary arteries, leading to an imbalance in the release of endothelial factors such as ET-1, PGI2 and NO, and PAECs undergoing mesenchymal transition [41]. Further mechanisms include reduced anticoagulant endothelial properties, increased expression of adhesion molecules (E-selectin, intercellular adhesion molecule 1, and vascular cell adhesion molecules), and release of different chemokines, cytokines, and growth factors. In addition, altered expression/function of ion channels and growth factor receptors, activation or inactivation of transcription factors such as nuclear factor of activated T cells (NFAT), hypoxia-inducible factor-1 (HIF-1), signal transducer and activator of transcription 3 (STAT-3), forkhead box protein O1 (FOXO1), and cellular metabolism dysregulation in PAECs, PASMCs, and fibroblasts all account for PH [41]. Finally, perturbed repairs in DNA and endothelial cell function are important for PH development [41]. Notably, HIF is a key regulator in the process of PH response, and the upregulation of HIF expression has been observed in patients with PH. The increased expression of HIF-1α is mainly derived from PASMCs, while HIF-2α is mainly derived from PAECs [42]. HIF-1α induces the expression of iNOS in lung tissue under hypoxia and then produces NO. Under physiological conditions, NO relaxes blood vessels by reducing the concentration of intracellular Ca^2+^. However, under hypoxic conditions NO produces cytotoxic effects, destroys the structure of vascular endothelial cells, promotes the proliferation of PASMCs, and promotes the contraction of pulmonary vessels [43].

## 4. Prostaglandins and PH

**Figure 1:**
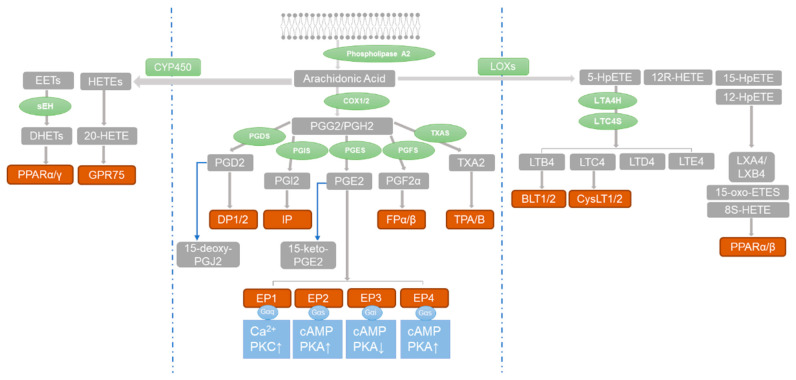
The three major metabolic pathways of arachidonic acid. Blue barrier lines are used to separate the pathways. Arachidonic acid is metabolized by enzymes (green) into compounds (gray) that activate receptors (red-brown). This activates G protein receptors (blue), leading to phosphorylation of downstream protein kinases (blue). The blue arrows in the figure indicate the conversion of PGD2 to 15-deoxy-PGJ2 and PGE2 to 15-keto-PGE2.

The prostaglandins (PGs) are a family of eicosanoids that can be synthesized from a number of essential fatty acids such as arachidonic acid (AA), docosahexaenoic acid (DHA), and eicosapentaenoic acid (EPA) [44]. They play important roles in pathophysiological processes such as inflammation, pain, fever, and tumorigenesis [45,46]. At present, many drugs targeting prostaglandin synthase, such as aspirin and celecoxib, are used to treat diseases. As shown in Figure 1, AA can be catalyzed into dozens of important lipid-active substances through at least three metabolic pathways: (1) the cyclooxygenase (COX) pathway catalyzes AA to prostanoids, including prostaglandin D2 (PGD2), PGI2, PGE2, and other prostanoids; (2) the lipoxygenase (LOX) pathway converts AA to leukotrienes (LTs); and (3) cytochrome P450 epoxygenses and P450 ω-hydroxylases catalyze AA to epoxyeicosatrienoic acids (EETs) and hydroxyeicosatetraenoic acids (HETEs) [47,48].

Numerous studies have shown that the metabolites of AA and its downstream pathways play important roles in PH. The key enzyme of the lipoxygenase pathway, 15-lipoxygenase and its metabolites 15-HETE, leukotriene (mostly LTB4) and eicosatrienoic acid (EET), participate in the progress of PH. In addition, maintaining the balance between local endothelium-derived PGs and LTs is critical for the homeostasis of the pulmonary vasculature [49,50,51,52,53]. Early studies found that PGI2, a metabolite of COXs, can reduce pulmonary artery pressure by promoting relaxation of pulmonary artery. At present, PGI2 analogues are the main means of clinical treatment of PH, although the efficacy of PGI2 analogues is not ideal [54].

Additionally, more and more studies have been carried out on the role of upstream key PG enzymes and other PG signaling pathways in PH. COX-1 is constitutively expressed in lung tissue. It has been reported that enhancing the activity of COX-1 in the trachea can ameliorate monocrotaline (MCT)-induced PH in rats [55]. COX-2 is an inducible enzyme regulated by growth factors and different cytokines such as interleukin (IL)-1β, IL-6, and tumor necrosis factor (TNF)-α [56]. Under chronic hypoxia, COX-2 is induced in the pulmonary vascular smooth muscle layer and catalyzes the formation of PGI2, which disrupts the balance between PGI2 and TXA2 [57]. These two products exhibit distinct roles in the vasculature. PGI2 is a vasodilator that inhibits platelet aggregation and thrombosis and inhibits proliferation, while TXA2 is a vasoconstrictor that induces platelet activation and aggregation and promotes proliferation [58]. Intra-arterial thrombosis has been reported in more than 60% of patients with hypoxia-related PH [59]. COX-2 gene deletion exacerbates PH, enhances sensitivity to TXA2, and induces intravascular thrombosis in response to hypoxia [57]. Consistent with previous observations, SC236, a selective COX-2 pharmacological inhibitor, has been found to reduce the production of PGI2 in a rat model of PH and to exacerbate pulmonary artery pressure elevation by increasing sensitivity to endogenous TXA2 while enhancing platelet activation [60]. In addition, studies have reported that COX-2 expression is upregulated in PH caused by congenital heart disease and that COX-2 has a protective effect on blood vessels and inhibits vascular remodeling [61,62]. Inhibition of COX-2 in healthy people and mice has been found to impair renal function while increasing blood pressure and thrombosis [63,64,65]. However, other studies have come to the opposite conclusion, finding that the selective COX-2 inhibitor celecoxib can reduce vascular tone by decreasing cAMP production, thereby preventing right ventricular pressure rise and improving MCT-induced PH. The authors speculated that the reason for this may be due to celecoxib improving PH by inhibiting the proliferation of PASMCs, just as celecoxib can play an anti-proliferative role by inducing apoptosis in cancer [66]. Studies have found that celecoxib can inhibit the proliferation of PASMCs induced by smoke extract by reducing COX-2-derived TXA2, resulting in an increase in the ratio of PGI2 to TXA2 and thereby improving COPD-induced PH [67]. Because COX-2 mediates the production of a variety of downstream prostaglandin products and inhibition of COX-2 can change a variety of downstream products, the role of COX-2 in PH remains to be further explored.

PGs, synthesized by COXs and different terminal synthases can exert their effects by binding to receptors involved in the process of pulmonary vascular remodeling. PGD2 exerts its biological function by binding to its receptors, namely, DP1 and DP2. DP1 is expressed in the human pulmonary artery and pulmonary vein, and activation of DP1 can induce pulmonary vascular relaxation [68]. Furthermore, DP1 improves pulmonary vascular remodeling in PH through PKA-mediated increase of mTORC1 activity [69]. TXA2 couples with the TP receptor to constrict pulmonary blood vessels, and TXA2 is significantly increased in the serum of PH, which is positively correlated with the severity of disease [70]. PGI2 plays a role in vasodilation and lowering blood pressure by activating IP, leading to the activation of adenylyl cyclase (AC) and increase of the intracellular cAMP level. Furthermore, IP is the target in the clinical treatment of PAH [71]. The role of PGE2 receptors in PH is described in the following section.

## 5. Role of PGE2 Receptors in PH

**Figure 2:**
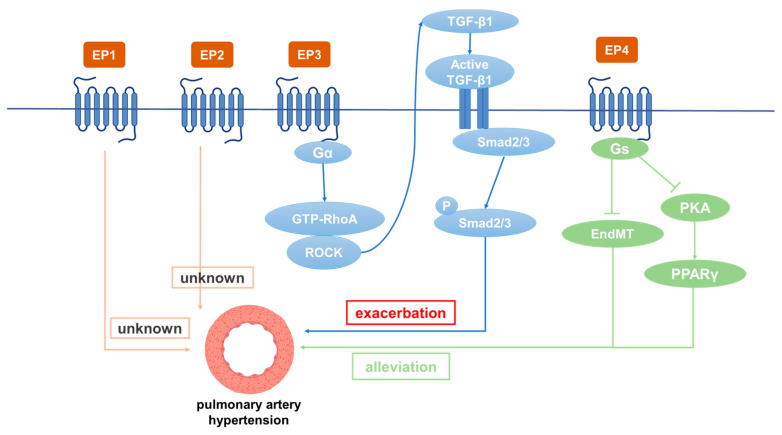
The role of different PGE2 receptors in the occurrence and development of PAH.

PGE2 is catalyzed by PGES and exerts its biological function by binding to EP receptors including EP1, EP2, EP3, and EP4. EP1 increases the intracellular Ca^2+^ level mainly by coupling with Gq protein. EP3 is usually coupled with Gi protein to inhibit intracellular cAMP level and PKA activity. Due to the existence of various isoforms, EP3 can be coupled with Gs to stimulate cAMP production and with Gq to stimulate the intracellular Ca^2+^ level. EP2 and EP4 increase intracellular cAMP levels by coupling Gs proteins and activating the PKA pathway. In general, PGE2 plays a critical role in blood pressure regulation. Its hypotensive effect is mainly achieved through EP2 and EP4, while activation of EP1 and EP3 raises systemic blood pressure [71]. Studies have shown that the COX/mPGES/PGE2/EPs system is essential for blood pressure regulation and vascular remodeling [72,73,74,75,76,77]. Studies have found that IP, EP3, and EP4 are highly expressed in normal pulmonary arteries, while EP2 is mainly located in the pulmonary veins [72]. Among the four EP receptors, EP3 and EP4 bind to PGE2 with the highest affinity (Kd < 1 nM), whereas EP1 and EP2 bind to PGE2 with low affinity (Kd > 10 nM) [73]. It has been found that PGI2 analogues both activate IP and act on EP receptors (Table 2). Many studies have revealed that different PGE2 receptors are involved in the occurrence and development of PH (Figure 2).

### 5.1. Role of EP1 in PH

It has been reported that oral administration of EP1 antagonist SC51322 reduces the blood pressure of spontaneously hypertensive rats. In addition, the systolic blood pressure of EP1 gene knockout mice was significantly lower than that of wild type mice, indicating that EP1 has the effects of constricting blood vessels and increasing blood pressure [77]. In a severe hypertension model, EP1 knockout was able to reduce blood pressure and alleviate organ damage [78]. In the pulmonary vein, EP1 counteracts the relaxation induced by PGs [79]. The selectivity of iloprost to different receptors is poor, and its effect of activating IP and EP1 is basically the same [80]. Iloprost has poor clinical efficacy, as it targets EP1 as well [81]. The EP1 antagonist SC-19220 inhibits the endocannabinoid arachidonyl ethanolamide (anandamide)-induced increase in pulmonary artery pressure [82]. Studies have shown that PDGF and VEGF promote abnormal proliferation and migration of ECs and SMCs to promote vascular remodeling, which can be reversed by the tyrosine kinase inhibitor imatinib in a dose-dependent manner [83]. Blockade of EP1/3 and TP or inhibition of the MAP2K, p38MAPK, PI3K-α/γ, and AKT/PKB signaling pathways prevented PDGF-induced contraction [84]. Due to the high contribution of the pulmonary venous bed to pulmonary vascular resistance, PDGF-BB-induced contraction is enhanced in the varicose veins of the pulmonary venous system [85]. Immunohistochemistry has shown that EP1 is mainly expressed in human pulmonary veins [86]. However, in PH patients and hypoxia-induced PH mice, EP1 expression did not change significantly [87]. Currently, the effect of EP1 on PH has not been reported.

### 5.2. Role of EP2 in PH

The expression of EP2 in PASMCs is upregulated in patients with PH [88]. Treprostinil, a drug currently used to treat PH, has high affinity for EP2 and IP [74] and increases cAMP content by activating EP2 in macrophages [23]. It is the only PGI2 analogue that can effectively bind to EP2, and the EP2 antagonist PF-04418948 (1 μM) significantly reduced the anti-proliferative effect of treprostinil [88]. In addition, studies have found that EP2 is associated with increased proliferation and migration of SMCs, all of which suggests that EP2 receptors have a protective role in vascular remodeling [89,90]. Treprostinil can significantly reduce the recruitment of fibroblasts at the site of vascular remodeling in hypoxic PH, and fibroblasts play a role in the inflammatory and proliferative phase of blood vessels [91]. Interestingly, EP2 expression in PASMCs was not affected in an MCT-induced rat PH model [92]. At present, the effect of EP2 on PH needs to be further explored.

### 5.3. Role of EP3 in PH

EP3 is widely expressed in the whole-body tissues of mice [93]. EP3 agonists have a strong contractile effect on isolated human pulmonary arteries [94]. The mean arterial pressure of EP3 knockout mice was found to be lower than that of wild type mice, suggesting that EP3 has the functions of constricting blood vessels and increasing blood pressure [95]. As the first stable oral PGI2 analogue, beraprost is mainly used in the clinical treatment of PH, arterial occlusive diseases, peripheral vascular diseases, renal failure, etc. [96]. Beraprost has been shown to improve exercise capacity and hemodynamics, thereby alleviating PH symptoms [97]. Other results have demonstrated that in addition to binding to IP, beraprost has a strong binding affinity with EP3 (Ki 110 Nm) in rats [23]. Many studies have provided evidence that the contractile effects of PGI2 analogues are mediated through EP3 receptors [75,98,99]. In patients with PH who were treated with beraprost but not selexipag (a prostaglandin receptor selective agonist), the vasodilator efficacy was reduced by the constriction caused by activation of EP3 in the pulmonary artery. In addition, a common side effect of beraprost is paradoxical constriction of the femoral artery due to activation of EP3 receptor. Therefore, patients with PH treated with PGI2 analogues experience leg pain, whereas selexipag is less likely to cause this side effect [100]. Esuberaprost, an isoform of beraprost, is five times more potent than beraprost in vasodilation of rat pulmonary arteries. Esuberaprost promotes cAMP production and inhibits proliferation of human PASMCs with inhibitory effects 40 times more potent than beraprost (EC50 3 nM and EC50 120 nM). The EP3 antagonist L-798106 can significantly reduce the pulmonary artery constriction effect of high concentrations of Esuberaprost. It is important to understand the role of EP3 in the contractile response, as this could limit the dose of PGI2 analogues provided therapeutically and potentially give rise to unwanted side effects [101]. In addition, EP3 plays a role in pulmonary vascular remodeling. Overexpression of EP3, especially its α and β isoforms, promotes the proliferation and migration of vascular SMCs, and EP3 knockout significantly improves vascular remodeling caused by a femoral artery guidewire strain [102]. Furthermore, EP3 expression has been found to be upregulated in hypoxia-treated PASMCs. The EP3 antagonist L-798106 ameliorated MCT- and hypoxia-induced PH and inhibited ECM deposition in pulmonary arteries. EP3 (mainly EP3α and EP3β) knockout in SMCs alleviated PH by inhibiting Rho/TGF-β1 signaling [87]. However, EP3-deficient mice have increased bleeding tendency [103]. Distal human PASMCs isolated from the pulmonary arteries (outer diameter: 1 mm) were found to be more susceptible to PGI2 analogue-induced proliferation inhibition than PASMCs isolated from the proximal pulmonary arteries (outer diameter: 0.8 mm) [104]. The expression of IP, EP3, FP, and TP in MCT-treated rats were all decreased compared with control rats (*p* < 0.05 or *p* < 0.01) [104]. Thus, EP3 is involved in the occurrence of PH, and its antagonists have therapeutic potential.

### 5.4. Role of EP4 in PH

EP4 plays a critical role in the closure of the ductus arteriosus at birth [105]. EP2 and EP4 have been reported to be the major mediators causing pulmonary vasodilation in rabbits [82]. The expression of IP, EP3, and EP4 in normal pulmonary arteries is much higher than EP1 and EP2. Patients treated with beraprost exhibited less disease progression at 6 months [106]. Additionally, it binds to EP4 and results in AC activation at lower affinity [107]. Levels of both PGI2 and PGE2 in plasma were dramatically depressed in experimental PH rats compared with controls. However, these depressed levels were elevated by beraprost treatment. Furthermore, both the dilatation response of vascular rings and the magnitude of the K_v_ channel response to beraprost were shown to be attenuated by the EP4 selective antagonist GW 627368X, suggesting involvement of EP4 in mediating the effects of PGI2 on O_2_-sensitive K_v_ channels and vasomotion [72]. While further studies are required to directly prove the interaction of beraprost and EP4, studies have reported that IP expression is significantly decreased in PH patients and rats, while the expression of EP4 is decreased slightly. The EP4 antagonist AH23848 can inhibit intracellular cAMP accumulation induced by iloprost in a dose-dependent manner, indicating that iloprost may mediate the diastolic function caused by EP4 instead of IP in PASMCs [92]. Cicaprost elevated cAMP in PASMCs four-fold compared with control, while iloprost only caused a one-fold increase [108]. This is probably because cicaprost has strong binding affinity to EP4 [23]. The PGE2-EP4 signal transduction pathway aggravates chronic inflammation and various autoimmune diseases. Therefore, specific antagonists for EP4 are expected to be effective therapeutic drugs for acute and chronic inflammation as well as for autoimmune diseases in non-pregnant adults [109]. Results have shown that reduced EP4 expression in macrophages can alleviate bleomycin-induced pulmonary fibrosis [110]. An increase in perivascular macrophages is essential in the development of hypoxia-induced PH in experimental animals [111]. Another study showed that EP4 knockout in mice increased airway inflammation induced by lipopolysaccharide (LPS) and cigarette smoke, while PGE2 inhibited the production of TNF-α and IL-6 in human lung macrophages by binding with EP4 [112,113]. SMC-specific EP4 knockout exacerbated angiotensin II-induced aortic dissection by increasing vascular inflammation [114]. PGE2 exerted anti-inflammatory effects by binding to EP4 to regulate macrophage and T lymphocyte functions, which are essential in innate and adaptive immunity as well as in tissue remodeling and repair. Evaluation of respiratory function is essential for patients with PH. For PH caused by COPD, inducing bronchial relaxation and reducing hypoxia may bring benefits to patients [115]. It has been found that EP4 agonists have a 10-fold to 50-fold greater bronchorelaxing effect than IP receptor agonists, and that PGE2-induced bronchiectasis is attenuated due to decreased expression of EP4 in PH associated with lung disease and/or hypoxia. Restoration of EP4 expression may be an effective way to improve the respiratory function of patients [116]. PGE2 inhibited PDGF-BB-induced proliferation and migration of human airway SMCs through EP4 to improve airway remodeling and improve COPD [117]. EP4 may be a new effective target for the treatment of PH. In addition, EP4 plays an important role in physiological and pathological vascular remodeling [114]. It was subsequently demonstrated that the expression of PPARγ in PAECs is decreased in PH patients [117] and that the loss of PPARγ in PASMCs or PAECs can cause pulmonary vascular remodeling, leading to PH and distal pulmonary artery muscularization [118]. L-902688, a selective EP4 agonist, has been reported to inhibit MCT-induced PASMC proliferation and migration as well as pulmonary vascular remodeling through PKA/PPARγ activation, which can ameliorate right ventricular fibrosis and TGF-β-induced endothelial–mesenchymal transition (EndMT) in PAH models [119,120]. Therefore, EP4 can inhibit the proliferation of PASMCs, improve pulmonary vascular remodeling, and suppress human lung macrophage inflammation, which is an important target for the treatment of PH [121].

## 6. Conclusions and Prospects

PGI2 and its analogues are potent vasodilators and possess antithrombotic and antiproliferative properties. All of these properties help to antagonize the pathological changes that take place in the small pulmonary arteries of patients with PH. In addition to IP, PGI2 analogues may present nonspecific binding to EP receptors, which may cause side effects and limit their efficacy. This review focuses on the role of different PGE2 receptors in the development of PH. In general, EP1 and EP2 expression are not affected in PH, and their specific role in PH remains unknown. PGE2 promotes the proliferation of SMCs through EP2, while the role of EP2 in PASMCs and PH needs to be further explored. The inhibition of EP3 (mainly EP3α and EP3β) can prevent the proliferation and migration of PASMCs and alleviate PH by inhibiting Rho/TGF-β1 signaling. EP4 activation can improve PASMC proliferation, pulmonary vascular remodeling, and right ventricular fibrosis while inhibiting EndMT. Therefore, this review reveals EP3 and EP4 as possible targets for the treatment of PH. 

## Figures and Tables

**Table 1:** Three classic drugs for the treatment of PH. The targets, therapeutic features, advantages, and disadvantages of these drugs and the preferences in different countries are compared in the table. PH: pulmonary hypertension; PAH: pulmonary arterial hypertension; ETAT: endothelial type A receptor; ETBR: endothelial type B receptor; PGI2: prostaglandin I2; PAP: pulmonary artery pressure; PVR: pulmonary vascular resistance; PDE5: phosphodiesterase 5 inhibitor.

Three Classic Drugs for the Treatment of PH	Classification	Drug Name	Administration Method	Target	Therapeutic Features	Advantages	Disadvantages	Preference
**1**	PGI2 analogues	Epoprostenol	Intravenous	IP	Slow, incremental and individualized dosing where the patient is closely monitored for tolerability. In most case, PGI2 analogues are reserved for patients with severe PH.	Exercise tolerance, hemodynamics, long-term survival and mortality of patients with PH has improved.	The half-life at room temperature is very short, requiring permanent intravenous catheter continuous infusion, causing infection and pain at the site of injection. It is complicated, uncomfortable for patients, and very costly. Common side effects: systemic hypotension, flushing, jaw pain and nausea. Its serious side effects: catheter associated sepsis.	Preferred drug; In North America and in some European countries since the mid-1990s
		Treprostinil	Subcutaneous, intravenous, inhalation and oral			A stable PGI2 analogue; Indexes of dyspnea, signs, symptoms and exercise capacity of PH, and hemodynamic measures significantly improve.	Causing infection and pain at the site of injection. Common side effects: systemic hypotension, flushing, jaw pain and nausea.	Alternative drug; In the United States since 2002
		Iloprost	Inhalation			A chemically stable PGI2 analogue; Hemodynamic values were significantly improved.	Its relatively short duration of action; It must be inhaled as many as 6 to 12 times a day; Side effects included cough and symptoms linked to systemic vasodilatation; It makes patients with PH have a higher rate of syncope.	In Japan
		Beraprost	Oral			The first biologically stable and orally PGI2 analogue which is absorbed rapidly; The peak concentration was reached 30 minutes after oral administration; With a half-life of 35–40 min.	There was no significant change in cardiovascular hemodynamics.	For treating primary PH in Europe.
	IP selective agonist	Selexipag	Oral			Specific for IP, it has little or no effect on other prostanoid receptors; The risk of the primary composite end point of death or a complication related to PAH was significantly improved.	Side effects: headache, diarrhea, systemic hypotension, flushing, jaw pain and nausea.	
**2**	Endothelin receptor antagonists	Bosentan	Oral	ETAR and ETBR	125 mg/bid Monthly monitoring of liver function tests is mandatory.	Significant improvements in PAP, cardiac output, and PVR	Development of abnormal hepatic function; It is contraindicated during pregnancy because of its teratogenic potential; Its long-term requires further evaluation.	For the treatment of PAH in North America in 2001 and in Europe in 2002.
**3**	Phosphodiesterase inhibitors	Sildenafil	Oral	PDE5		long-term adjunctive treatment can improve exercise capacity and pulmonary hemodynamics.	The experience with sildenafil is preliminary, and controlled studies are in progress to determine its efficacy, side effects, and safety.	

**Table 2:** IP and EP1–4 binding affinities (Ki) for PGI2 analogues in human and mouse. Radioligand binding data (Ki in nM) are from original study references for PGI2 analogues [73,74,75,76]. Blank means Ki value > 3 μM, ND means not done, and YES indicates evidence for functional activity.

PGI2 Analogues		IP	EP1	EP2	EP3	EP4
Iloprost	Human	4	1	1172	203	212
	Mouse	11	21	1600	27	2300
Treprostinil	Human	32	212	3.6	2505	826
	Mouse	YES	ND	YES	ND	ND
Beraprost	Human	39			680	
	Mouse	16			110	
Cicaprost	Human	17	>1340	>1340	255	44
	Mouse	10	1300		170	
